# Glycated albumin precipitation using aptamer conjugated magnetic nanoparticles

**DOI:** 10.1038/s41598-020-67469-6

**Published:** 2020-07-01

**Authors:** R. Fayazi, M. Habibi-Rezaei, M. Heiat, F. Javadi-Zarnaghi, R. A. Taheri

**Affiliations:** 1 School of Biology, University of Tehran, P.O.Box 14155-6455, Tehran, Iran; 2 Center of Excellence in Nano-Biomedicine, University of Tehran, Tehran, Iran; 3 Research Center for Gastroenterology and Liver Disease, Baqiyatallah University of Medical Sciences, Tehran, Iran; 4 Department of Cell and Molecular Biology and Microbiology, Faculty of Biological Science and Technology, University of Isfahan, Isfahan, Iran; 5 Nanobiotechnolology Research Center, Baqiyatallah University of Medical Sciences, Tehran, Iran

**Keywords:** Nanobiotechnology, Biochemistry, Biotechnology, Diseases

## Abstract

To develop a strategy for the elimination of prefibrillar amyloid aggregates, a three-step non-modified DNA aptamer conjugation on silica-coated magnetic nanoparticles was carried out to achieve aptamer conjugated on MNP (*Ap-*SiMNP). Prefibrillar amyloid aggregates are generated under a diabetic condition which are prominently participated in developing diabetic complications. The binding properties of candidate DNA aptamer against serum albumin prefibrillar amyloid aggregates (*AA20*) were verified using electrophoretic mobility shift assay (EMSA) and surface plasmon resonance spectroscopy (SPR) analysis. The chloro-functionalized silica-coated MNPs were synthesized then a nano-targeting structure as aptamer conjugated on MNP (*Ap-*SiMNP) was constructed. Finally, *Ap-*SiMNP was verified for specific binding efficiency and *AA20* removal using an external magnetic field. The candidate aptamer showed a high binding capacity at EMSA and SPR analysis (*K*_*D*_ = 3.4 × 10^─9^ M) and successfully used to construct *Ap-*SiMNP. Here, we show a proof of concept for an efficient bio-scavenger as *Ap-*SiMNP to provide a promising opportunity to consider as a possible strategy to overcome some diabetic complications through specific binding/removal of toxic *AA20* species.

## Introduction

Magnetic nanoparticles (MNPs) are the most promising nano-materials with a broad range of medical applications as theranostics (mutual applications in therapy and diagnosis) due to their mutually enjoying from the high surface to volume ratios and superparamagnetism properties beside on their nontoxicity and biocompatibility^1^. One of the most health-threatening approaches is the generation of toxic morph aggregates of proteins/peptides as amyloid structures which are also progressively generated upon protein glycation under diabetic conditions. These structures not only can be toxic by themselves but also play a role as a seed to enhance and spread the amyloidogenesis process to bring about neuropathies, retinopathies, nephropathies and so on^2^. It has been reported that MNPs negatively interfere with the generation of amyloid structures^3^. Numerous studies have demonstrated that MNPs in conjunction with capturing agents such as antibodies or aptamers, enjoying bio-specificity and magnetism, have a great potential to bio-specifically bind to and also remove target species. Aptamer conjugated MNPs (*Ap*-MNP) that have been used for proteins and cell detection^4^, antibody labeled MNPs for Aβ detection in blood and in vitro^5,6^ and capturing of amyloid β and tau protein from the human whole blood^7^ are some examples that can be taken into account. Aptamers have been reported for β-sheet-rich fibrillar amyloid assemblies of amyloidogenic proteins/peptides such as full-length PrP^8^, fibrillar β2-microglobulin^9^, α-synuclein oligomers^10^, oligomeric Aβ40^11^, Aβ42 protofibrils^12^ and even amyloidogenic product of advance glycated end product of serum albumin (albumin-AGE)^38^. Although aptamer functionalized magnetic nanoparticles has been reported for capturing salivary cortisol in a sensing platform-specific to salivary cortisol^13^ or nanocomposite of gold nanoparticles with aptamer for specifically binding to A*β* oligomers by an antibody-aptamer sandwich assay^14^, there has been few or no report dealing with the proof of concept for the synthesis and application of ssDNA aptamer conjugation on MNPs toward glycated albumin removal.

Aptamers (aptus; fit, and meros; part) are single-stranded DNA (ssDNA) or RNA (ssRNA) molecules, which specifically bind to a target molecule through attaining a specific three-dimensional structure^15^. From 1980 to 2017, the number of people (age 18–99 years) diagnosed with diabetes (including mostly 5–10% types 1 and 90–95% type 2 diabetes mellitus; T1DM and T2DM, respectively) worldwide have experienced a rate of 8.6% annual increase from 108 to 451 million, which is expected to rise at lowered 2% rate of annual increase to 693 million by 2045^16^. Besides, the mortality rate of people with diabetes has doubled from 1990 to 2010^17^. In hyperglycemic conditions, protein glycation occurs in which reducing sugars non-enzymatically react with α- and ε-primary amino groups of protein to reversibly generate Schiff base, then irreversibly rearrange to form Amadori products known as advanced glycation end products (AGE)^18^. Protein glycation results in conformational changes enough to make proteins prone to be aggregated in a manner known as amyloidogenesis in which native protein structures change to soluble cross-beta structures (prefibrills) and follow to form insoluble amyloid fibrils^19^, with a featured geometry in spite of the protein type^20^.

Glycation modifies several proteins in the circulatory system including albumin, as the most abundant protein in plasma^21^ from a friend to foe^22^. Serum albumin plays a central role in a wide variety of physiological and pharmacological mechanisms, including contribution to the maintenance of redox balance in the extracellular fluids^23,24^. About 65% of the serum albumins are at the reduced form with a redox thiol group on Cys-34 residue (HSA-SH)^24^ which in connection with the dominant presence in the circulation, albumin accounts for 80% of plasma free thiols^25–27^. Increased glycation of serum albumin in diabetes alters its properties, especially antioxidant function^28^. The ratio of glycated albumin (GA) in the health condition range between 1 and 10%, while at the diabetic condition it may rise two or three folds and even achieve to 90%^26^. GA plays an important role in diabetic complications through a multi-ligand receptor as a receptor of advanced glycated end products (RAGE)^29^ which participates in oxidative stress, inflammation, activation of nuclear factor kappa B (NF-κB) and ROS formation^30^.

Glycation products especially as toxic soluble prefibrills are involved in amyloid disorders, including Alzheimer (AD), Huntington (HD), Parkinson (PD) and T2DM^20,23,31^. Therefore, not only their formation should be prevented but also there is a serious demand to find effective strategies to eliminate them. In this regard, prevention of albumin glycation or its neutralization has been mentioned as an effective strategy to decrease diabetic complication^26^. Clements has shown that neutralization of glycated albumin by specific monoclonal antibody can alleviate diabetic retinopathy^32^. Consequently, it is highly likely that removing of GA and toxic soluble GA prefibrillar spices can bolster diabetes mediated complications. Allocating the burden of elimination responsibility to MNPs conjugated targeting molecules, can positively impact on elimination of GA and toxic soluble GA prefibrillar spices from solution.

On the whole, finding a concept for recognition of toxic soluble GA prefibrillar spices by developing a novel strategy for DNA aptamer conjugation on silica-coated MNP (*Ap-*SiMNP) and precipitation of GA amyloids using *Ap-*SiMNP under an external magnetic field, has been targeted in the present report.

## Results

In the present report, the *Ap*-SiMNPs was synthesized through a novel strategy described in the materials and methods section, then used to precipitate the soluble and toxic glycated albumin species. The serum albumin amyloid formation was induced by glycation and monitored by Thioflavin T (ThT) fluorescence emission. ThT has an affinity for cross beta rich structures which brings about its excitation at 420 nm with maximum emission intensity at around 488 nm^33^.

**Figure 1:**
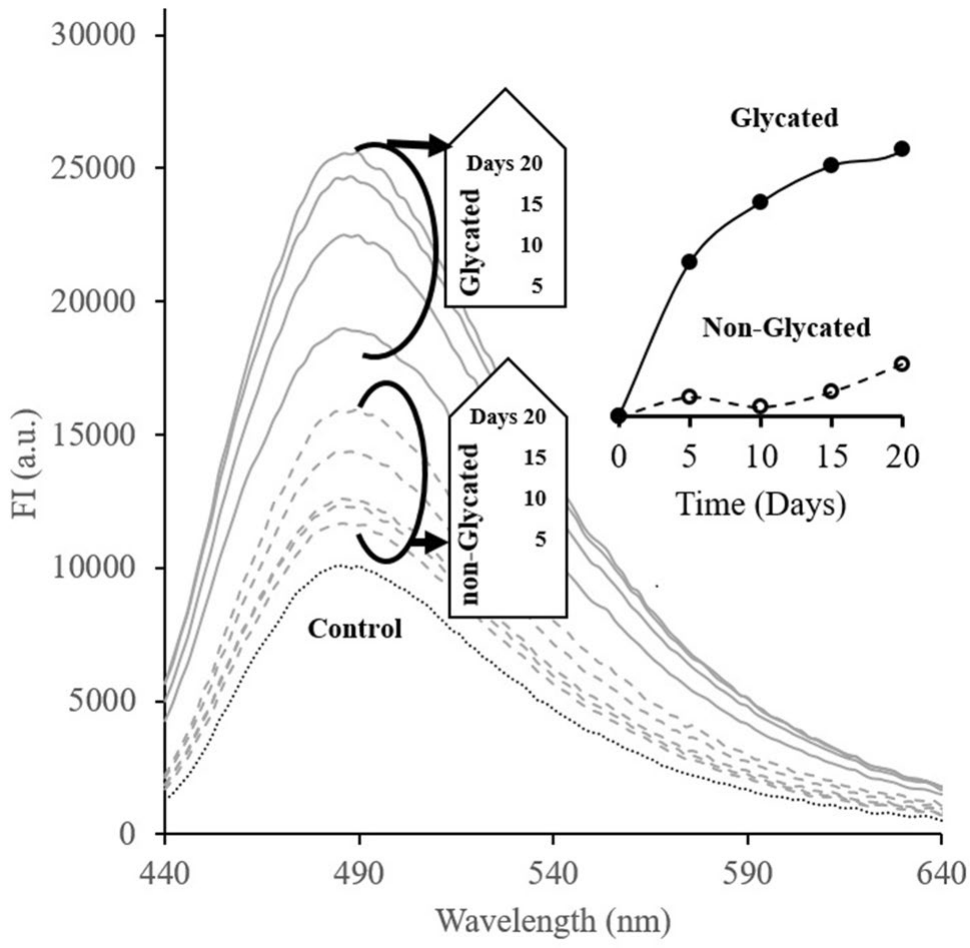
ThT fluorescence emission spectra (λ_ex_ = 420 nm) of incubated albumin in the absence or presence of fructose displayed as non-glycated and glycated, respectively. ThT fluorescence emission intensity (λ_em_ = 488) changes in non-glycated and glycated samples over incubation time are demonstrated as the inset to figure.

ThT results revealed the formation of beta rich structures through serum albumin glycation for up to 20 days. As depicted in Fig. 1, the ThT fluorescence intensity increases with time of albumin incubation in the presence of fructose (Fig. 1). Therefore, to achieve toxic prefibrillar soluble amyloid species, the incubation was carried out up to 20 days when fluorescence emission intensity almost reaches a plateau at 488 nm without the formation of insoluble species in glycated protein samples.

**Figure 2:**
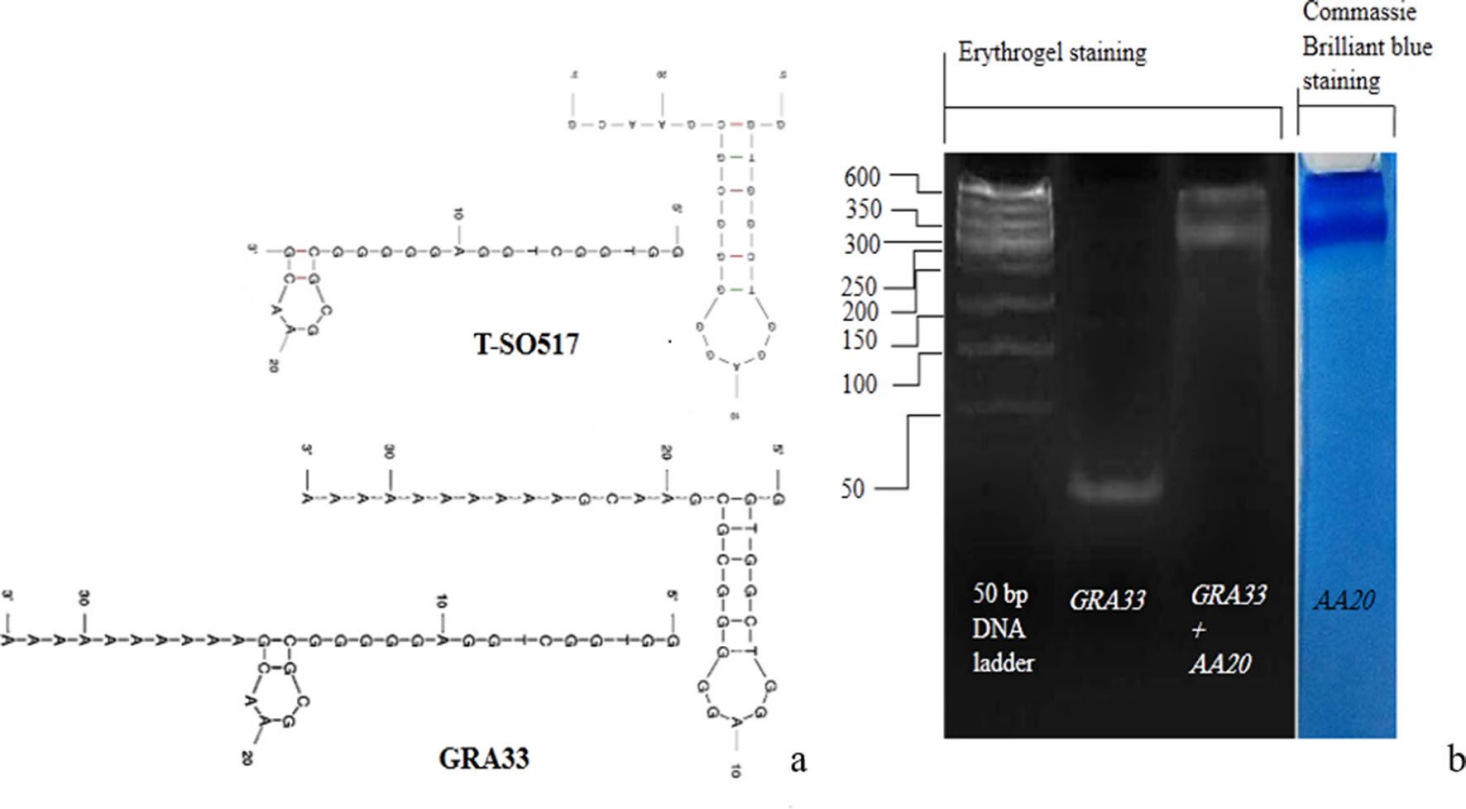
(**a**) Mfold predicted-structures for both of *T-SO517* and *GRA33* aptamers in 0.15 M Na^+^ at 25 °C generated by mfold web server (https://unafold.rna.albany.edu/?q=mfold/DNA-Folding-Form). (**b**) EMSA of *GRA33* alone and incubated with *AA20* protein. The mobility of free *GRA33* and *GRA33–AA20* stained by Erythrogel safe stain has been shown. The *AA20* within the same gel has been cut down and stained with Coomassie blue is presented in the right lane.

In the case of verification of modified aptamer (*GRA33*) conformation, the structures of *T-SO517* and *GRA33* were predicted using the mfold web server^34^. Both aptamers folded to form similar one stem-loop secondary structures, suggesting that the poly A tail at 3ʹ of *GRA33* does not interfere in the secondary structure of T-SO517 (Fig. 2a). The poly A tail helps aptamer to fold properly after conjugating to MNPs surface without space exclusion effect. The result of the electrophoretic mobility shift assay (EMSA) confirms that *GRA33* can selectively recognize and bind to *AA20* while it is not able to bind to the hemoglobin as negative control. As depicted in the Fig. 2b, *GRA33* mobility in the absence of *AA20* is faster than 50 bp in marker but *GRA33* incubated with *AA20* was retarded in native PAGE stained by Erythrogel DNA safe stain, to make it present in the *AA20* region, documented with the same gel stained by Coomassie Brilliant Blue.

**Figure 3:**
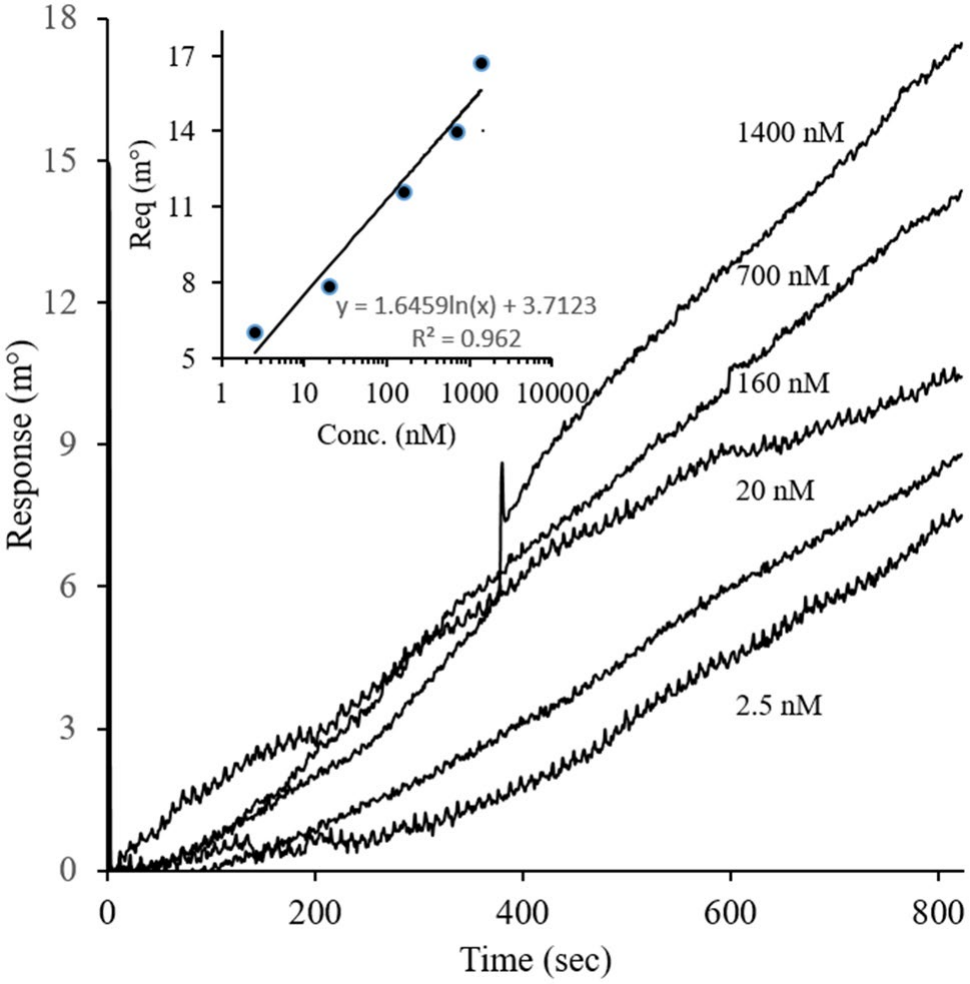
SPR Sensogram of *GRA33–AA20* interaction. Different concentration of *GRA33* (2.5, 20, 160, 700 and 1,400 nM) was loaded on immobilized *AA20* on the sensor chip. The equilibrium responses were also plotted as a function of the aptamer concentration and fitted to a 1:1 Langmuir binding model to determine the dissociation constants.

Surface plasmon resonance (SPR) assay was also conducted to confirm the binding of the aptamer to *AA20* and to determine the aptamer dissociation constant (*K*_*D*_). Figure 3 shows the responses of the sensor surface bypassing different concentrations of the aptamer. Sensogram showed a higher response to the higher concentration of the aptamer. These responses were fitted for simple 1:1 binding showing the apparent *K*_*D*_ = 3.4 × 10^−9^ M.

**Figure 4:**
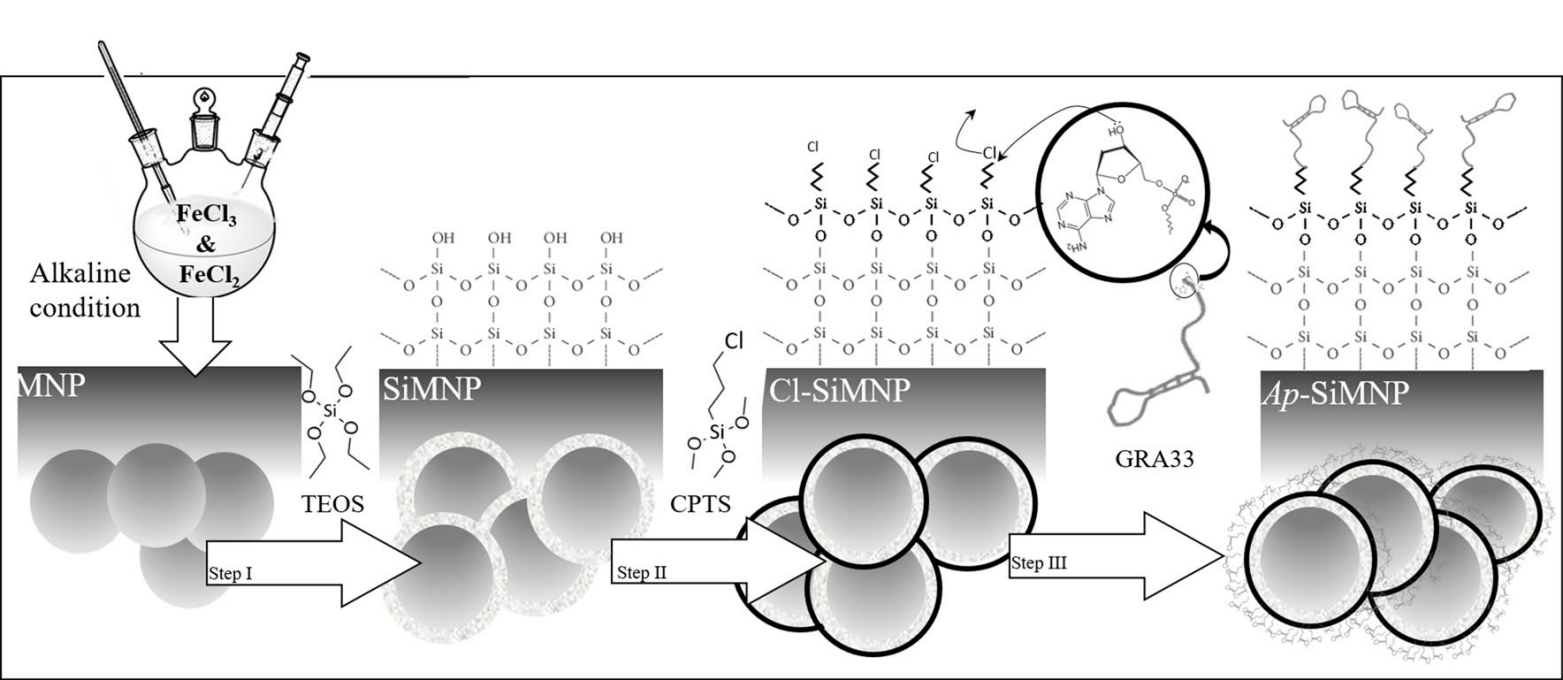
Schematic representation of the applied three-step protocol of the non-modified aptamer/MNP conjugation (the detail has been provided in “Materials”), [generated using Microsoft PowerPoint 2013 (15.0.4569.1504) MSO (15.0.4569.1506) 64-bit and in part using Microsoft Paint, Version 1903 (OS Build18362.592).

A novel three-step strategy applied for non-modified DNA aptamer/MNP conjugation to achieve conjugated aptamer on MNP as summarized in Fig. 4. The Fe_3_O_4_ magnetic nanoparticles (MNPs) prepared by the chemical co-precipitation method were conjugated by candidate DNA aptamer to achieve *Ap*-SiMNPs. Accordingly, MNP was coated with silica (SiO2) to synthesis silica coated MNP (SiMNP) using tetraethyl orthosilicate (TEOS) under acidic condition followed by its chloro-functionalization using a solution of chloropropyltrimethoxy silane (CPTS) under dry condition (for details see methods). At the final step, the non-modified aptamer (*GRA33*) conjugation on chloro-functionalized SiMNP (Cl-SiMNP) was performed through a nucleophilic substitution mechanism in which chlorine is a leaving group (for details see methods). The carbon–chlorine bond in the substituted chloropropyl moieties has weaker average bond enthalpy (338 kJ mol^−1^) than carbon–carbon (348 kJ mol^−1^) or carbon–hydrogen (412 kJ mol^−1^). Moreover, this bond is polar which makes it more reactive and as a result provided slightly positive carbon atom prone for nucleophilic attack by the hydroxyl group in the deoxy-ribosyl moiety of 3′end of the GRA33 through a single step bimolecular S_N_2 mechanism.

**Figure 5:**
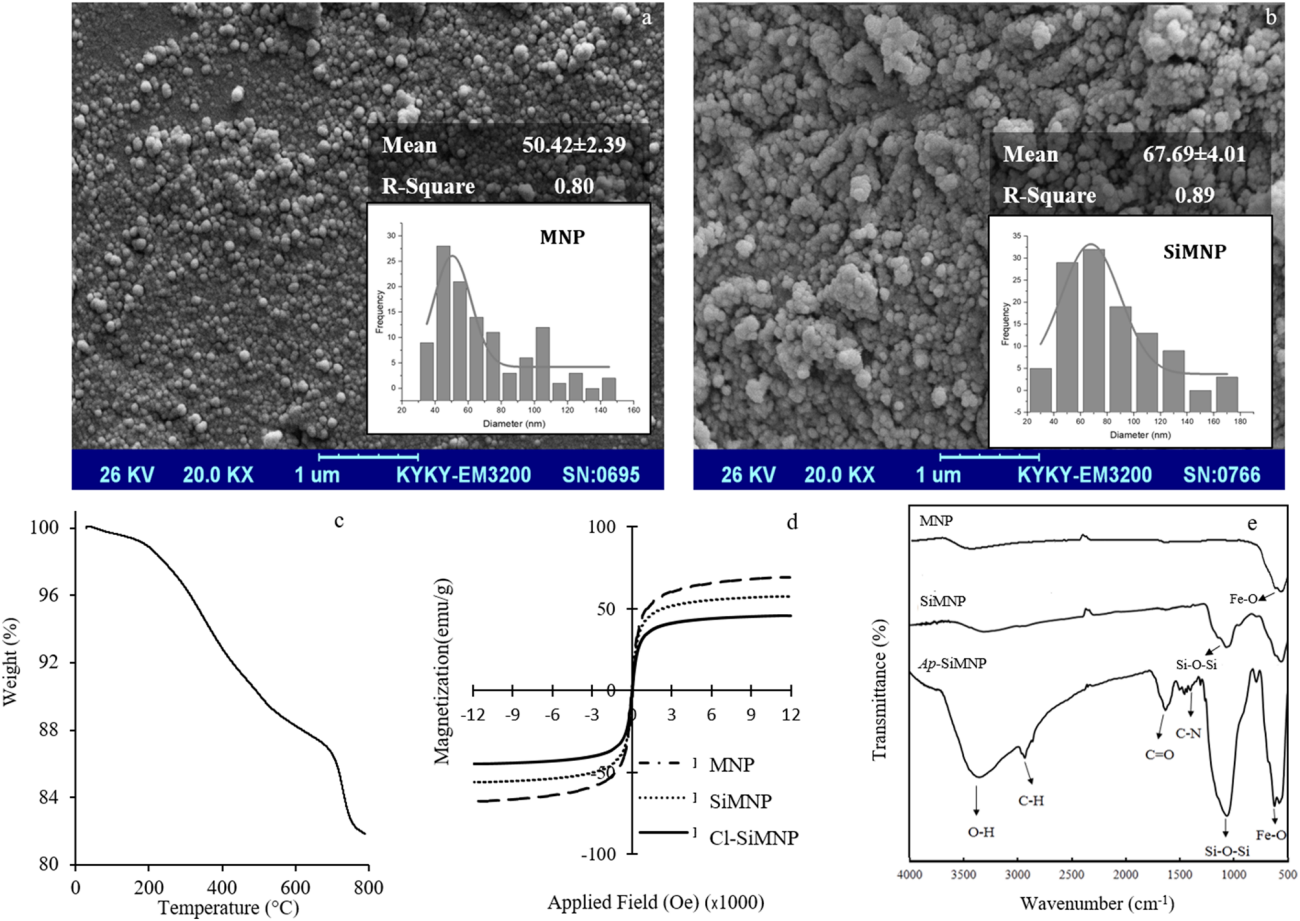
Scanning electron microscopy (SEM) image of MNP (**a**) and SiMNPs (**b**).The insets of the figures show the size distribution plots. (**c**) TGA analysis of Cl-SiMNPs and (**d**) VSM magnetization curve of MNP, SiMNP and Cl-SiMNP. (**e**) FTIR spectra of the nanoparticles (MNP, SiMNP and *Ap-*SiMNP).

**Figure 6:**
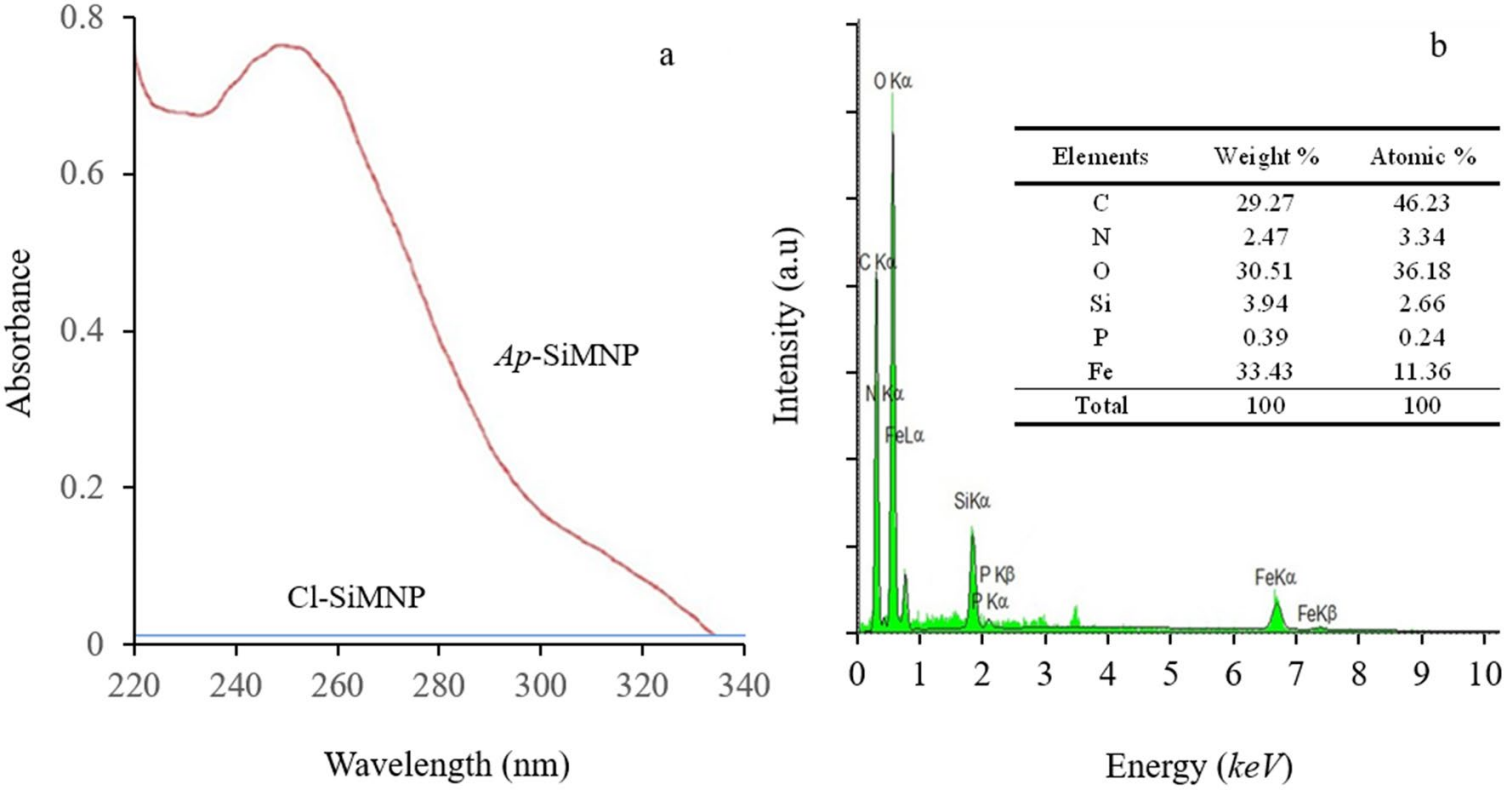
(**a**) UV absorbance spectrum of Cl-SiMNPs and *Ap*-SiMNPs by Nanodrop, (**b**) EDX spectrum of *Ap*-SiMNPs (elemental composition has been shown as inset table).

The results regarding the characterization of the modified magnetic nanoparticles are shown in Figs. 5 and 6. Figure 5a,b shows the spherical shapes and related size distribution plots of magnetic nanoparticles before and after coating with silica as MNP and SiMNP at mean sizes of 50.42 ± 2.39 and 67.69 ± 4.01 nm, respectively. Thermal gravimetric analysis (TGA) can measure the amount and the rate of organic compound mass changes as a function of time and temperature by raising the temperature here up to 600–800 °C (Fig. 5c). In this study, the mass changes of Cl-SiMNPs were monitored to confirm the binding of CPTS to SiMNP. As shown in Fig. 5c, the first step of weight loss that occurred up to 200 °C was related to the loss of water in the sample and the second step of weight loss in 200–700 °C was related to thermal decomposition of the organic compound. As a result, ⁓ 12.5% of weight loss, is equivalent to ⁓ 0.81 mmol chloropropyl per milligram nanoparticle covalently attached to SiMNPs surface. In continue, the VSM analysis have been performed on Fe_3_O_4_ (MNP), Fe_3_O_4_@SiO_2_ (SiMNP) and Fe_3_O_4_@SiO_2_@CPTS (Cl-SiMNP) nanoparticles (Fig. 5d). As shown, nanoparticles represented super-paramagnetic properties however, the silanization then further coating with chloropropyltrimethoxy silane (CPTS) decreased the magnetization of particles. Moreover, the synthesis and modification of all magnetic nanoparticles were confirmed by FTIR spectroscopy (Fig. 5e). The characteristic bands at 580 and 634 cm^−1^ were indications of Fe–O stretching and characteristic bands at 803 and 1,096 cm^−1^ were related to Si–O–Si and Si–OH, respectively, which confirmed the formation of silica-coated magnetic nanoparticles (SiMNPs). Substitution of chloropropyl groups on the MNPs produced Cl-SiMNPs which were verified by stretching vibrations band at 2,900 cm^−1^ in FTIR spectra, based on aliphatic C–H groups as the characteristic of propyl group. Finally, in the case of *Ap*-SiMNP synthesis*,* *GRA33* conjugation on Cl-SiMNPs was confirmed by the presence of stretching vibrations band at 1,350 and 1696 cm—^1^ which were related to aromatic C–N and C=O groups of DNA aptamer, respectively.

Spectroscopic analysis of nanoparticles, including Cl-SiMNPs and *Ap*-SiMNPs, were performed in the range of 220–340 nm) Fig. 6a). *Ap*-SiMNPs spectrum presents a strong absorbance pick at 260 nm while Cl-SiMNPs have not similar nucleic acid representative absorbance pick which confirm successful attachment of single-stranded DNA aptamer molecules on SiMNPs to result in *Ap*-SiMNPs. Besides, to confirm the *Ap*-SiMNP conjugation, the chemical composition of *Ap*-SiMNPs was analyzed using EDX (Fig. 6b). The energy dispersive X-ray (EDX) spectrum shows the signal peaks of elements, including phosphor (P), nitrogen (N), carbon (C), iron (Fe) and silica (S). Phosphor (P) and nitrogen (N) are indicative of the chemical elements for aptamer in *Ap*-SiMNPs.

**Figure 7:**
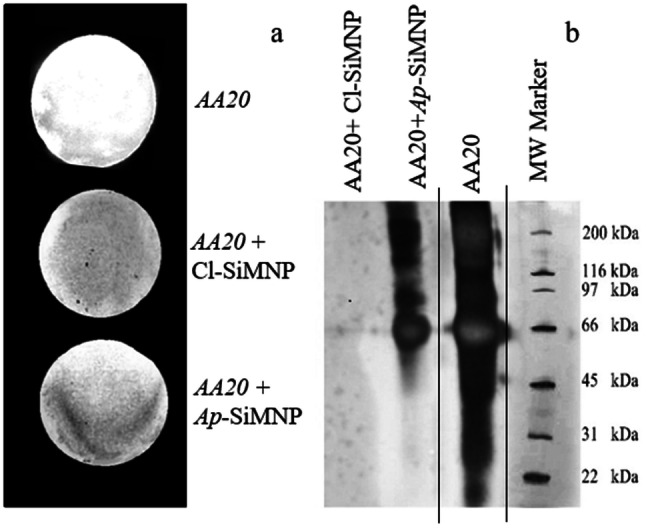
(**a**) Dot blot pattern for colorimetric detection of three blots of *AA20* on nitrocellulose membranes after exposing with TBS buffer (as control), Cl-SiMNPs and *Ap-*SiMNPs in the same buffer. (**b**) The result for SDS-PAGE of *Apta-*precipitation analysis to detect the AA20 eluted from *Ap*-SiMNPs and Cl-SCMNPs as test and control, respectively. The enclosed part by black lines is from different part of the same gel. The MW marker has been included from another gel electrophoresis with the same condition. Full-length gel is presented in SI Fig. S4. The experiment was performed using 12% acrylamide gel at 80 V for 2.5 h, and then stained by silver nitrate.

The binding capacity of the *Ap*-SiMNPs to *AA20* was investigated using dot blot analysis and Apta-precipitation analysis as shown in Fig. 7. Dot blot analysis was performed to verify the specific binding capacity of constructed *Ap-*SiMNP to *AA20* in comparison to Cl-SiMNPs*.* Figure 7a shows three *AA20*-blotted nitrocellulose membranes after exposing separately with TBS buffer (as control), Cl-SiMNPs and *Ap-*SiMNPs in the same buffer in a dot blot analysis. Accordingly, the only membrane exposed with *Ap*-SiMNP has bound to the *AA20* blots while in the absence of nanoparticles (control) or presence of Cl-SiMNP, specific binding is not observed. Moreover, the specific apta-precipitation of *AA20* by *Ap*-SiMNPs using a magnetic field was carried out to present specific capturing capability for constructed *Ap*-SiMNPs. The precipitated *AA20* captured by *Ap*-SiMNPs was eluted by urea then analyzed using SDS-PAGE (Fig. 7b). Although *Ap*-SiMNPs has successfully captured *AA20* as depicted band at 65 kDa, but Cl-SiMNPs as control has failed to precipitate *AA20* that no protein corresponding band is seen in the same region.

## Discussion

T2DM becomes one of the biggest threats to human health^17,35^. In spite of recommended clinical management for T2DM, so far, no definitive therapy has ever known for this disease^36–38^.

In T2DM, hyperglycemia leads to glycation of albumin and other plasma proteins to generate soluble β-rich protein aggregates as the main player in developing diabetic complications which finally form insoluble amyloid fibrils^39,40^. The evolution of dedicated targeting molecules such as antibodies and aptamers provides effective tools to confront non-communicable diseases including Alzheimer’s disease (AD) and other diabetic complications^41^. For instance, an antibody against amyloid-beta (Aβ) to detect Aβ protofibrils in biological samples^42^ or Aβ derived diffusible ligands (ADDL) specific antibody for AD diagnosis^43^ have been reported using enzyme-linked immunosorbent assay (ELISA) or MNP conjugated specific antibody, respectively. However, too much effort has been recently devoted to using aptamers with their stunning capabilities as the next-generation targeted therapeutics^44^. Yusuke Kaida et al*.* in 2013 designed an aptamer against albumin-AGE for therapeutic purposes and showed that aptamer administration decreases the albumin-AGE toxicity thereafter nephropathy in diabetic mice^45^.

Retrospectively, nanoparticles have great potential in accompaniment with targeting molecules such as aptamers to reinvigorate their power. NP constructs conjugated by aptamer has already been employed in bio-separation methods. Herr et al*.* in 2008 employed aptamer conjugated MNPs for the detection and separation of cancer cells^46^. Delaviz et al*.* in 2015 also used aptamer conjugated MNPs in order to separate the Hepatitis C virus from plasma^47^. Tsukakoshi et al*.* developed T-SO517 aptamer for α-synuclein oligomers which also was capable to recognize and bind to amyloid-beta oligomers with the same affinity^10^. However, aptamer-nanoparticle construct preparation has been remained to be a fundamental pillar in diagnostic and therapeutic strategy designing in Nanomedicine. Various strategies have been employed to join aptamers to the NP and most of them depend on using aminated or biotinylated aptamers^48,49^. However, here we are reporting a novel three-step strategy of the non-modified aptamer/MNP conjugation to achieve conjugated aptamer on MNP (Fig. 4). The spheroid shape of nanoparticles beside on silica coating of the MNPs toward generating SiMNP core–shell structures have been confirmed by SEM images and related size distribution data in Fig. 5a,b. Accordingly, silica coating on MNPs, brought about the increasing in the mean size values from 50.42 ± 2.39 to 67.69 ± 4.01 for MNP and SiMNP, respectively.

According to the thermogram presented as TGA in Fig. 5c, 0.81 mmol chloropropyl per milligram nanoparticle covalently attached to SiMNPs surface. The chlorine on the surface of the magnetic nanoparticle as anionic leaving group makes it susceptible and more prone to make a chemical reaction with hydroxyl and amine groups^50–52^.

**Figure 8:**
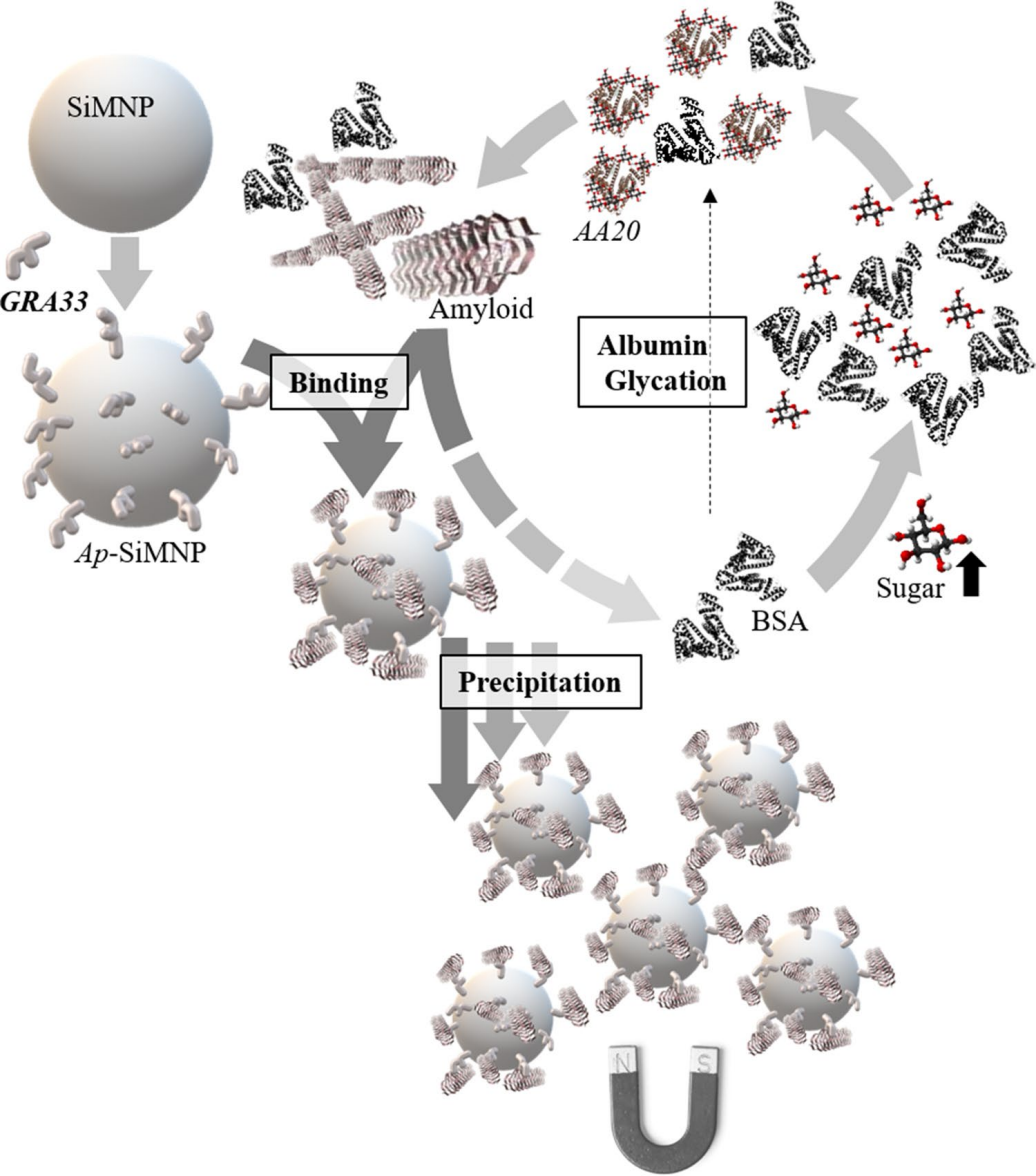
Schematic representation of bio-specific magnetic precipitation of glycated albumin species using conjugated aptamer (*GRA33*) on Cl-SiMNP [generated using Microsoft PowerPoint 2013 (15.0.4569.1504) MSO (15.0.4569.1506) 64-bit and in part using Microsoft Paint, Version 1903 (OS Build18362.592)].

Hence, covalent bond between aptamer and nanoparticle is expected by playing chlorine as a leaving group. The VSM results indicate that MNP, SiMNP and Cl-SiMNP nanoparticles had retained the super-paramagnetic properties at room temperature (Fig. 5d). The magnetization saturation values of MNP, SiMNP, and Cl-SiMNP resulted as 69.16, 57.16 and 45.80 amu g^−1^, respectively. However, the decline in magnetization saturation values indicates the silica coating and successful functionalization by CPTS. Moreover, silica coating and aptamer conjugation on silica coated magnetic nanoparticles have been documented using FTIR spectroscopy (Fig. 5e) then have been supported by aptamer representative peak at 260 nm by UV spectroscopy and EDX analysis (Fig. 6). The conjugation product is strong enough to be employed in nano-biomedicine approaches. As a result, the loading density of the aptamer onto the surface of the activated particles as Cl-SiMNPs was achieved to be at 0.1 mmol g^−1^ nanoparticle. The functionality of the conjugation product was approved through the *Apta*-precipitation and Dot blot analysis (Fig. 7) which also indicates preservation of a desired aptamer conformation even after conjugation process. As summarized in Fig. 8, here, we are reporting bio-specific precipitation of prefibrillar GA species as *AA20* by conjugated aptamer *GRA33* on Cl-SiMNP using an external magnetic field as a potential strategy toward relieving T2DM complications.

All data of EMSA (Fig. 2), SPR (Fig. 3) and dot blot (Fig. 7) well confirmed that *GRA33* is capable to identify and bind to prefibrils, before or after conjugating to MNPs and supports the previous hypothesis which explains amyloid moieties exhibit similar structural features regardless of their primary sequences^20,53^. The resulted *K*_*D*_ value at 3.4 × 10^−9^ M represents high-affinity interaction between *GRA33* and *AA20.* Moreover, the achieved 0.1 mmol g^−1^ aptamer loading density onto the magnetic nanoparticle will make it possible to precipitate 0.1 mmol AA20 per gram of *Ap*-SiMNPs.

Through an apta-precipitation method, collectively, we were able to separate the GA from a sample under an external magnetic field upon apta-precipitation strategy, employing superparamagnetic nanoparticle in conjugation with GRA33. The reference range of serum GA is 11.9–15.8% (currently, GA unit clinically is %)^54^, which was converted to 218–287 mmol mol^−^1 using a conversion formula that converts GA (mmol mol^−1^) into GA (%) as “GA (%) = 0.05652.GA (mmol mol^−1^)—0.4217”^55^]. Moreover, considering that the reference concentration of serum albumin is 50 g L^−1^ or 0.75 mM, the normal serum GA concentration range will be 0.163–0.215 mM. Therefore, it will be theoretically possible to remove whole serum GA using about 8–10 g of *Ap*-SiMNPs, which is remained to be explored. Our findings suggest that hiring nano-sized tools in combination with targeting molecules like *Ap*-SiMNPs provides a promising opportunity to overcome amyloidosis-related complications in non-communicable chronic diseases.

These tools are on a similar scale to biomolecules like proteins that are involved in chronic and metabolic diseases. So, they can deal with them very well and can also be controlled in a magnetic field. The mfold predicted structures are based on a possible base-pairing event, while they can be more complicated in vitro through the forming of complex structures^56^. However, there are still many ambiguous aspects of using aptamers as therapeutics and in order to explore how this aptamer folds and works in the real situation, more efforts should be devoted to investigating in vivo features. In conclusion, the novel non-modified aptamer conjugation on MNP to synthesize *Ap*-SiMNPs nano-construct provides promising results and prospects the capability to scavenge toxic albumin amyloid species from solution and has merit to be considered as a therapeutic tool in order to prevent or decrease diabetic complications and other amyloid disorders.

## Methods

### Material

Bovine serum albumin, fructose and all reagents and chemicals were purchased from Merck. SYBR Green was purchased from Thermo Fisher Scientific., Erythrogel DNA safe stain was prepared from Arash Teb Pishro Pars., Thioflavin T (ThT) purchased from Fluka and single stranded DNA (ssDNA) Aptamer T-SO517^10^ with poly A tail (10 nt) at the 3ʹ end (*GRA33*) was synthesized by SinaClon.

### Preparation and evaluation of prefibrillar amyloid aggregates

Prefibrillar amyloid aggregates were prepared using serum albumin under glycation condition. Albumin (0.75 mM) in 0.1 M phosphate buffer pH 7.2 was incubated in sterile condition at 37 °C in the absence or presence of 500 mM fructose. Aliquots were taken at 5 days’ time intervals up to day 20. The amyloid aggregates formation was studied by ThT fluorescence intensity in non-glycated and glycated serum albumin samples according to Ferdousi et al*.*^2^*,* whereby the prefibrillar albumin amyloids (AA) at day intervals up to day 20 designated as *AA0*, *AA5,* *AA10,* *AA15,* *and* *AA20*, were excited at 42 nm and their emission spectra from 440 to 660 nm was recorded by synergy H1 Multi-Mode Microplate Reader (BioTek). The data represent the average of three independent measurements.

### Aptamer binding analysis

The *GRA33* was synthesized and its secondary structure was predicted and illustrated using mfold web server (https://unafold.rna.albany.edu/). *GRA33* specificity to prefibrillar albumin was analyzed using electrophoretic mobility shift assay (EMSA)^57^. Aptamer was heated to 90–100 °C for 5 min and immediately cooled on ice for 5 min to avoid DNA mega-clamp and to achieve the correct three dimensional (3D) structure^58,59^. *GRA33* at 1 mM was solubilized in 10 µL buffer, including Tris–HCl 10 mM, NaCl 150 mM, KCl 5 mM, pH 7.4 and concentration was adjusted using UV–Vis spectrophotometer (NanoDrop-2000, Thermo Fisher Scientific). Samples as *GRA33*, *AA20,* *GRA33*+*AA20* and *GRA33*+BSA (as control) were run on native PAGE (10%) at 80 V for 90 min, then were visualized using Erythrogel DNA specific dye using UV gel doc and Coomassie Brilliant Blue for aptamer and protein, respectively. Moreover, binding affinity and dissociation constant (*K*_*D*_) of *GRA33* to *AA20* were determined using surface plasmon resonance spectroscopy (SPR) (Autolab ESPRIT, Eco Chemie B.V, the Netherlands)^60^. *AA20* (50 µg/mL) was covalently attached to the sensor chip surface and different *GRA33* concentrations in the range of 2.5–1,400 nM were passed through it. Dissociation constant (*K*_*D*_) was determined using equilibrium response (R_eq_) for each concentration using Langmuir isotherm for 1:1 binding state by manufacturer software (kinetic evaluation software version 5.4).

### Preparation of the *Ap*-SiMNP

Fe_3_O_4_ magnetic nanoparticles (MNPs) were prepared by chemical co-precipitation method under alkaline condition using FeCl_3_·6H_2_O and FeCl_2_·4H_2_O. Then, silica (SiO2) was coated on MNPs to synthesis silica coated MNP (SiMNP) using tetraethyl orthosilicate (TEOS) and glycerol under acidic condition (pH 4.6) according to Vojoudi et al*.*^61^*.* In order to prepare aptamer-conjugated magnetic nanoparticles (*Ap*-SiMNP), a solution of chloropropyltrimethoxy silane (CPTS) (4.5 mmol) was added to the suspension of 1 g SiMNP in 50 mL dry toluene and then, it was refluxed at 112 °C for 24 h. The resulting chloro-functionalized SiMNP (Cl-SiMNP (were washed by toluene and dried at room temperature. Then, the solutions of 0.1 mmol *GRA33* and 1 µmol triethylamine were sequentially added to the suspension of 1 g Cl-SiMNP in 4 mL absolute ethanol and refluxed at 70 °C for 21 h. The loading density of aptamer onto the surface of the Cl-SiMNP was differentially achieved by subtracting the mole numbers of unbound aptamer from initially incubated aptamer in the presence of the activated NPs using UV absorbance at 260 nm. The resulting *Ap*-SiMNPs were washed thrice with deionized water, then were precipitated using magnetic field.

### Characterization of magnetic nanoparticles

Fourier transform infrared spectroscopy (FTIR) (*WQF-510*) measurements were performed to analyze Fe_3_O_4_ nanoparticle and surface characterization of every step from silica coating to aptamer-functionalization. The scanning electron microscopy (SEM) images were examined using an SEM system (*CamScan* *MV2300* with an acceleration voltage of 26 keV and a magnification of 70,000) to study the morphology and size of MNPs. In continue, the size distribution analysis were carried out using *ImageJ* and *Origin* *pro* *2016* softwares. The magnetic property of the synthesized nanoparticles was analyzed by vibrating sample magnetometer (VSM, Meghnatis Daghigh Kavir Co.; Kashan Kavir; Iran). Thermal gravimetric analysis (TGA) was performed through heating the sample (Cl-SiMNPs) from room temperature to 800 °C at a rate of 10 °C min^−1^ under argon flow using a thermal gravimetric analyzer (TGA Q50 V6.3 Build 189). Energy dispersive X-ray spectroscopy (EDX) (*TESCAN* *Vega* *Model*) was also performed in order to identify aptamer-SiMNP conjugation by analyzing elements of *Ap*-SiMNPs. Ultimately, the UV spectra analysis (from 220 to 340 nm) was performed by UV–visible spectrophotometer (Nanodrop-2000, Thermo Fisher Scientific) for Cl-SiMNPs and *Ap*-SiMNPs in order to verify the conjugation process.

### Dot blot assay

The binding capacity of *Ap*-SiMNPs to *AA20* was evaluated by dot blot assay. According to dot blot standard protocol^62^. Two blots of *AA20* (0.75 mM) were placed on each of three nitrocellulose membranes. Then, membrane surfaces were blocked using 3% BSA in 5% TBST (Tris-buffered saline and Tween 20) (Tris–HCl 20 mM, pH 7.5, NaCl 150 mM, Tween 20 0.05%) to prevent nonspecific interactions. In continue, one *AA20* blotted membrane was taken as blank and each of two others was treated with suspensions of 1 mg ml−^1^ Cl-SiMNPs or *Ap*-SiMNP in TBS buffer (Tris–HCl 20 mM, pH 7.5, NaCl 150 mM). Blank membrane was separately treated with TBS buffer (without nanoparticles). Membranes were washed with TBS buffer and dried at room temperature.

### Prefibrillar amyloid precipitation

The ability of *Ap*-SiMNPs to precipitate *AA20* as glycated albumin was tested using the apta-precipitation method^63^. 10 µL of *Ap*-SiMNPs (1 mg mL^−1^) were incubated with 5 µL of *AA20* (200 µM) at room temperature for 45 min under gentle stirring. Incubated *Ap*-SiMNPs were separated using magnetic field and the supernatant was discarded. *Ap*-SiMNP sediment was washed five times by 0.1 M phosphate buffer pH 7.2 to remove nonspecific trapped species. Then 20 µL of urea (8 M) was added to each sample to elute bound proteins from *Ap*-SiMNPs. Again, *Ap*-SiMNPs were magnetically precipitated and supernatant was run on SDS-PAGE (12%) at 80 V for 2.5 h. All steps were done by Cl-SiMNPs as negative control. The bands on the gel were visualized using the silver staining method.

## Supplementary information

Supplementary information
