# Supplementary material for: Glycated albumin precipitation using aptamer conjugated magnetic nanoparticles
Source: Sci Rep. 2020 Jul 1;10:10716. doi: 10.1038/s41598-020-67469-6 (PMC7329883; doi:10.1038/s41598-020-67469-6)
Supplement: Supplementary file 1 — Supplementary information [file 41598_2020_67469_MOESM1_ESM.docx]

**Supplementary**

**Glycated Albumin Precipitation using Aptamer Conjugated Magnetic Nanoparticles**

R. Fayazi^a^, M. Habibi-Rezaei^a, b*^, M. Heiat^c^, F. Javadi-Zarnaghi^d^, R A. Taheri^e^

^a^ School of Biology, University of Tehran, Tehran, Iran

^b^ Center of Excellence in Nano-Biomedicine, University of Tehran, Tehran, Iran.

^c^ Research Center for Gastroenterology and Liver Disease, Baqiyatallah University of Medical Sciences, Tehran, Iran.

^d^ Department of Cell and Molecular Biology & Microbiology, Faculty of Biological Science and Technology, University of Isfahan, Isfahan, Iran.

^e^ Nanobiotechnolology Research Center, Baqiyatallah University of Medical Sciences, Tehran, Iran.

***Corresponding author**

School of Biology, University of Tehran, Tehran, Iran

*P.O.Box* *14155-6455. Tehran, Iran.*

<Tel:+98-21-61113214>, Fax: +98-21-66971941

E-mail: [*mhabibi@ut.ac.ir*](mailto:mhabibi@ut.ac.ir) *, mhabibirezaei@gmail.com*


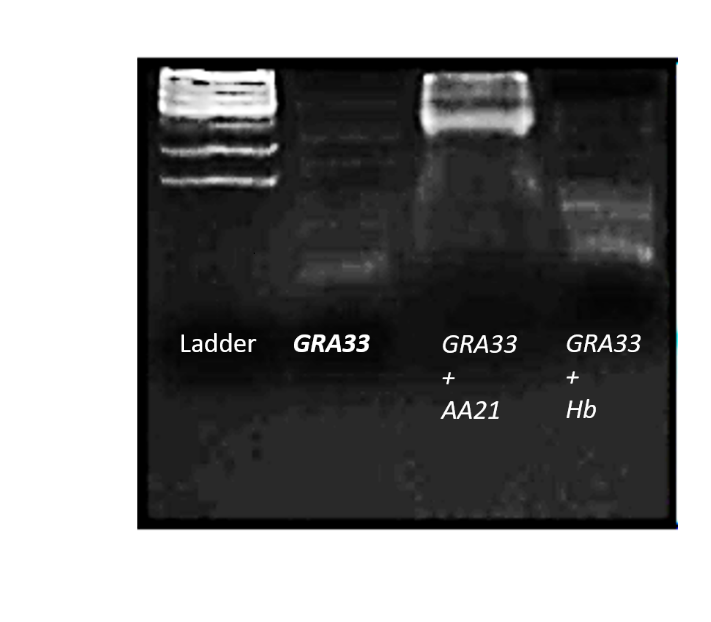


**Supplement 1.** EMSA of GRA33 alone and incubated with albumin amyloid aggregate from day 21 (AA21) protein as well as hemoglobin (Hb) as negative control to show that GRA33 is able to selectively bind AA20 but do not bind to Hb.


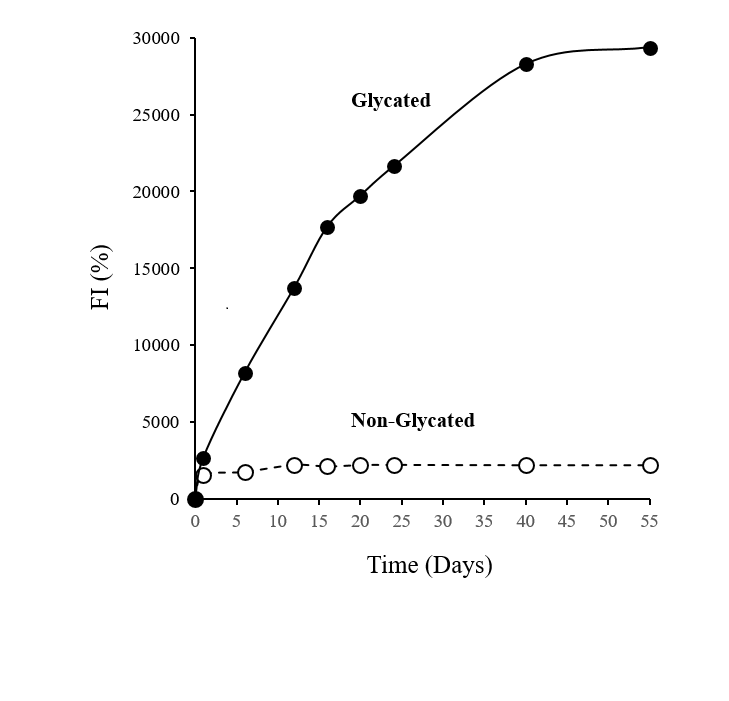


**Supplement 2.** ThT fluorescence emission spectra (λ_ex_=420 nm) of incubated albumin for in the absence or presence of fructose for 55 days, displayed as non-glycated and glycated, respectively. ThT fluorescence emission intensity (λ_em_=488) changes in non-glycated and glycated samples over incubation time are demonstrated as the inset to figure.


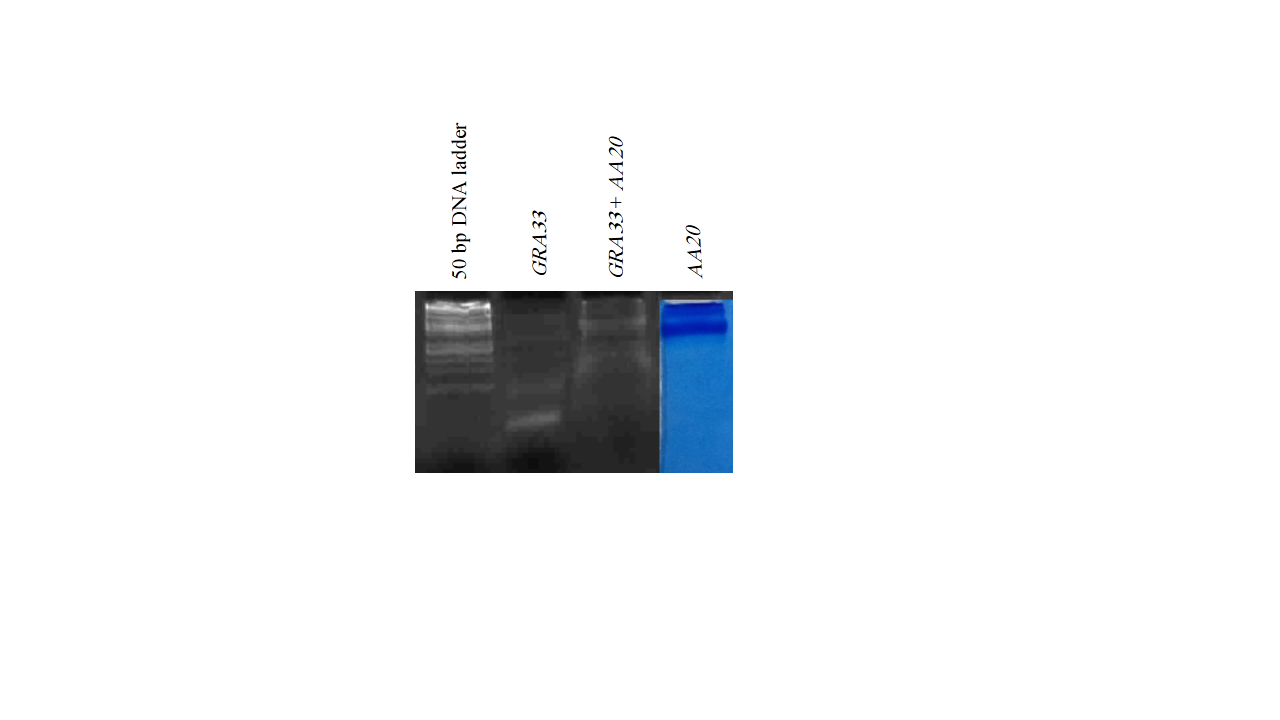


**Supplement 3**. The full length of EMSA gel (*GRA33* alone and incubated with *AA20* protein before contrast/brightness adjustment), supporting the figure 2a.


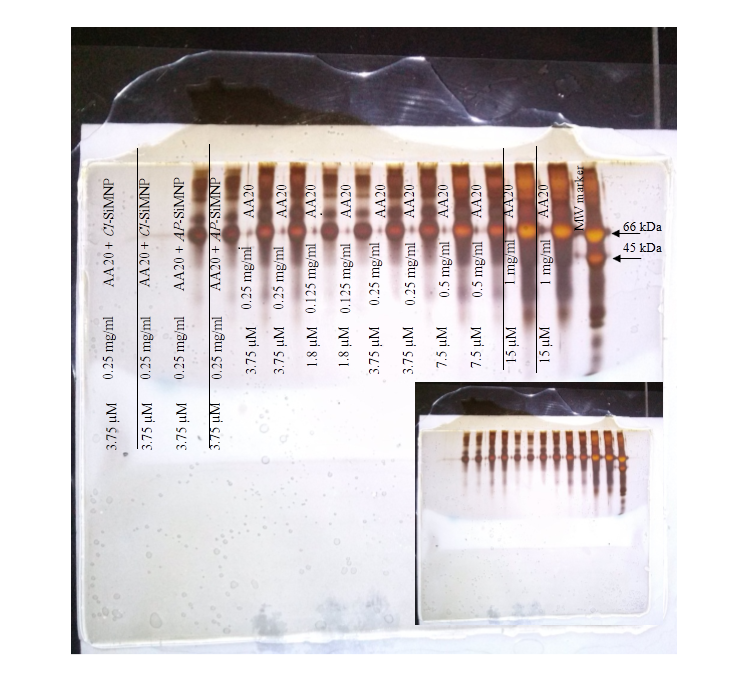


**Supplement 4**. The full SDS-PAGE (*Apta-*precipitation analysis to detect the AA20 eluted from *Ap*-SiMNPs and Cl-SCMNPs as test and control all at 15 μM, respectively), supporting the figure 7b, before brightness adjusting. The enclosed regions by black lines are cropped and used in figure 7b. The experiment was performed using 12% acrylamide gel at 80 V for 2.5 h, then stained by silver nitrate.

*
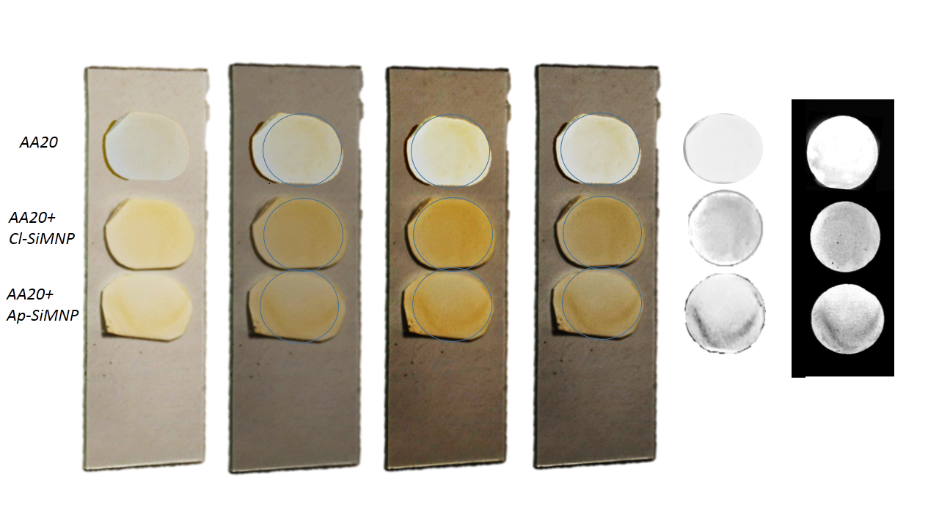
*

**Supplement 5**. The Original and serial cropping, contrast/brightness adjustments on dot blots. Supporting the figure 7a.
